# Improving the error rates of the Begg and Mazumdar test for publication bias in fixed effects meta-analysis

**DOI:** 10.1186/1471-2288-14-109

**Published:** 2014-09-22

**Authors:** Miriam Gjerdevik, Ivar Heuch

**Affiliations:** Department of Mathematics, University of Bergen, P. O. Box 7800, N-5020 Bergen, Norway; Department of Global Public Health and Primary Care, University of Bergen, P. O. Box 7804, N-5018 Bergen, Norway

**Keywords:** Meta-analysis, Publication bias, Rank correlation, Kendall’s tau, Spearman’s rho, Error rates

## Abstract

**Background:**

The rank correlation test introduced by Begg and Mazumdar is extensively used in meta-analysis to test for publication bias in clinical and epidemiological studies. It is based on correlating the standardized treatment effect with the variance of the treatment effect using Kendall’s tau as the measure of association. To our knowledge, the operational characteristics regarding the significance level of the test have not, however, been fully assessed.

**Methods:**

We propose an alternative rank correlation test to improve the error rates of the original Begg and Mazumdar test. This test is based on the simulated distribution of the estimated measure of association, conditional on sampling variances. Furthermore, Spearman’s rho is suggested as an alternative rank correlation coefficient. The attained level and power of the tests are studied by simulations of meta-analyses assuming the fixed effects model.

**Results:**

The significance levels of the original Begg and Mazumdar test often deviate considerably from the nominal level, the null hypothesis being rejected too infrequently. It is proven mathematically that the assumptions for using the rank correlation test are not strictly satisfied. The pairs of variables fail to be independent, and there is a correlation between the standardized effect sizes and sampling variances under the null hypothesis of no publication bias. In the meta-analysis setting, the adverse consequences of a false negative test are more profound than the disadvantages of a false positive test. Our alternative test improves the error rates in fixed effects meta-analysis. Its significance level equals the nominal value, and the Type II error rate is reduced. In small data sets Spearman’s rho should be preferred to Kendall’s tau as the measure of association.

**Conclusions:**

As the attained significance levels of the test introduced by Begg and Mazumdar often deviate greatly from the nominal level, modified rank correlation tests, improving the error rates, should be preferred when testing for publication bias assuming fixed effects meta-analysis.

**Electronic supplementary material:**

The online version of this article (doi:10.1186/1471-2288-14-109) contains supplementary material, which is available to authorized users.

## Background

Meta-analysis is a systematic procedure for assessing and combining statistical information based on results of available independent studies regarding the same topic. In recent years, meta-analytic methods have become increasingly popular in various fields of medicine. Results from meta-analysis are subject to criticism for many reasons, an important concern being possible small study effects such as publication bias. Publication bias arises when the published studies relevant for inclusion in a meta-analysis do not represent all studies of the problem of interest [1].

In particular, studies that are less likely to get published appear to be the less conclusive ones [2, 3]. The chance that studies with small sample size and low statistical precision are published is increased if they show stronger treatment effects [4, 5]. Publication bias may affect the conclusions of meta-analyses and systematic reviews and result in a biased overall estimate of the treatment effect. Hence data should be evaluated for publication bias before a meta-analysis is conducted [6].

Traditionally, funnel plots [7], a simple graph of the measure of precision (e.g. sample sizes or inverse variances) of the component studies versus the summary outcome measures, have been used as a visual tool to detect small study effects. The funnel graph is based on the fact that precision in estimating the underlying effect will increase as the sample size of the component studies increases [8]. The estimated effects should in principle be symmetrically distributed about the true unknown effect. Results from studies with small sample size should scatter widely at the bottom of the graph, with the spread narrowing as the sample sizes increase. Assuming that all studies in the analysis estimate the same effect, the plot should resemble a symmetrical inverted funnel if publication bias is not present. If publication bias is present, however, the plot will tend to be skewed, due to the fact that small and non-significant studies are less likely to appear in the published literature. This induces a correlation in the graph.

Several authors have provided formal and objective tests for publication bias. Egger et al. [8] based their test on a simple linear regression of the effect estimate against its standard error, weighted by the inverse of the variance of the effect estimate. A modified regression approach was introduced by Macaskill et al. [4]. Begg and Mazumdar [6] exploited the fact that publication bias will tend to induce a correlation between the treatment effects and their variances. They constructed a test by examining the correlation between the two factors and standardized the effect sizes prior to performing a rank correlation test based on Kendall’s tau.

Asymmetry in funnel plots may also, however, occur due to heterogeneity. Statistical heterogeneity is present when the true effects being evaluated vary between studies, and this underlying heterogeneity may be detectable if the variation between the studies is above that expected by chance [9]. The proposed tests for publication bias actually test for small study effects. They seek to assess whether funnel plot asymmetry is likely to have occurred by chance and are thus not able to distinguish between small study effects such as publication bias and heterogeneity. However, as we will conduct simulations studies of meta-analyses using the fixed effects model, which assumes no heterogeneity, we will often use the less precise phrase “publication bias”. The meaning should be clear from the context.

**Figure 1:**
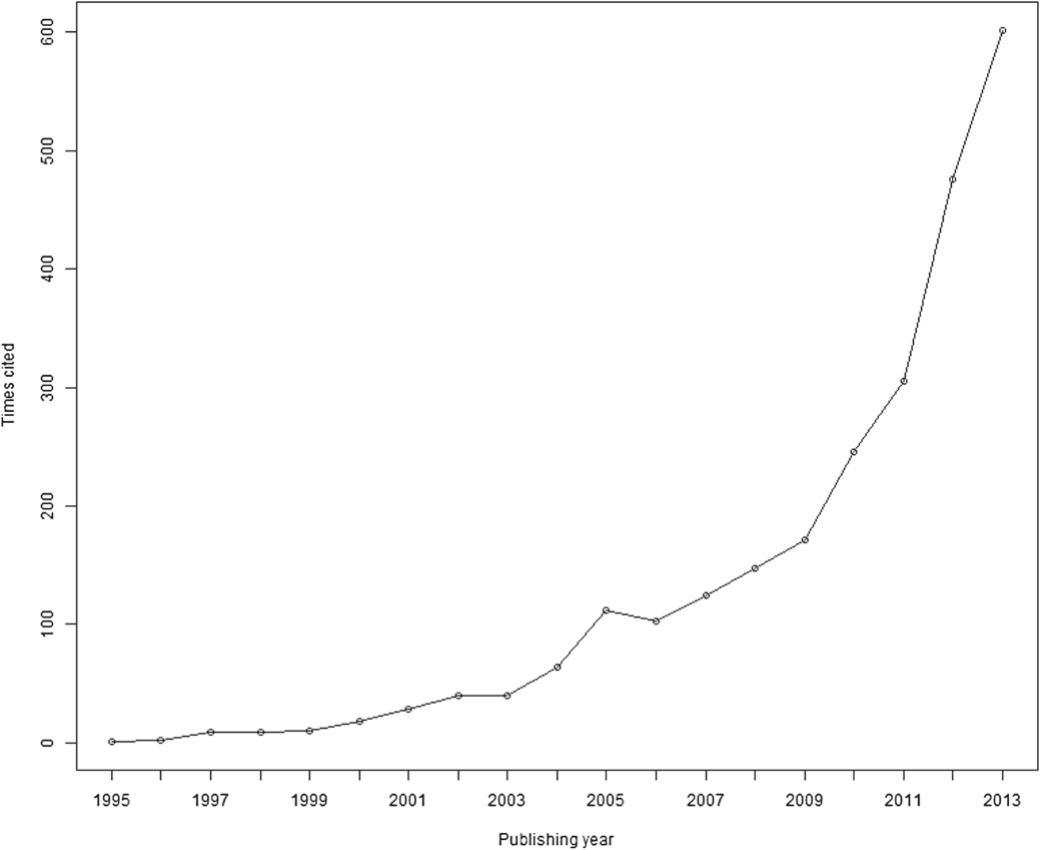
**Number of yearly cites for the Begg and Mazumdar article.**

The regression based approach proposed by Egger et al. [8] and the rank correlation test provided by Begg and Mazumdar [6] are widely used in meta-analysis to test for small study effects in clinical and epidemiological studies. As of December 2013, the original article by Egger et al. [8] had been cited more than 7,000 times in the Web of Science database [10]. Still, the article by Begg and Mazumdar [6] had been cited more than 2,500 times, a number which demonstrates great influence in the literature. During 2013, the number of new citations exceeded 600. Figure 1 illustrates the increasing impact and relevance of the rank correlation test almost two decades after it was published, although it is not quite so important as the Egger test [8].

Concerns have been expressed, however, about the possible lack of power of both tests [4, 6]. It is also well-known that when the outcome is binary and the intervention effect is expressed as an odds ratio or a relative risk, the variance of the estimator is mathematically related to the estimate itself [11, 12]. This results in the null hypothesis being rejected too frequently, and several authors have proposed alternative tests to correct for this bias [5, 13, 14]. Nonetheless, concentrating on the rank correlation test, it is still an open question whether the significance level is satisfactory in the case of a continuous outcome variable. Improving the error rates of the Begg and Mazumdar test for publication bias in meta-analysis, assuming a normally distributed outcome, is thus a primary objective of our article.

The significance level of the Begg and Mazumdar test is attained when there is no selection bias present in the meta-analysis. Begg and Mazumdar carried out simulations corresponding to such situations. They did not, however, include the results in their paper but merely stated that “In all cases the nominal significance level was less than 5%” [6]. This remark should be interpreted with caution, but probably indicates that the significance level of the test is less than the nominal 5% level. Nonetheless, the issue of the significance level warrants further investigation.

### Description of the Begg and Mazumdar test

Suppose that a meta-analysis consists of *k* studies. Let *t*_1_,*t*_2_,…,*t*_*k*_ and *v*_1_,*v*_2_,…,*v*_*k*_ denote the estimated effect sizes and sampling variances from these studies. As the effect sizes are not identically distributed under the null hypothesis of no publication bias, Begg and Mazumdar [6] standardize the effect sizes prior to performing a rank correlation test. They correlate  and *v*_*i*_, *i*=1,2,…,*k*, where


Here,


is the standard weighted average of the effect sizes and


is the variance of .

Begg and Mazumdar [6] use a rank correlation test based on Kendall’s tau, defined as


The test involves evaluating *C*, the number of pairs of studies that are ranked in the same order with respect to the two factors ***t***^***∗***^ and ***v***, and *D*, the number of pairs of studies that are ranked in the reverse order. Here ***t***^∗^ and ***v*** denote the vectors consisting of  and *v*_1_,*v*_2_,…,*v*_*k*_, respectively. The standardized test statistic is defined as


a variable which is asymptotically *N*(0,1), a direct result following from the properties of Kendall’s tau [15]. It is also possible to employ an exact distribution of Kendall’s tau for small values of *k*, although this is not done by Begg and Mazumdar. The denominator should be modified if there are tied observations [15].

This article is organized as follows. In the Methods section we suggest an algorithm intended to improve the error rates of the Begg and Mazumdar test for publication bias. Additionally, this section outlines the simulation procedure used to study and compare the new algorithm to the original test in fixed effects meta-analysis. In the Results section we explain why the Begg and Mazumdar method has a poor significance level. The performance of the adjusted test is assessed and compared to the results of the original test. Examples are given. The Discussion section includes an overall evaluation of the rank correlation tests for publication bias presented in our paper and is followed by the Conclusion section.

## Methods

### Improvement of the Begg and Mazumdar test: Method and algorithm

We would like to develop a test based on rank correlation making it easy to adjust the actual significance level in the case of a normally distributed outcome variable. This can be done employing the simulated distribution of the estimated measure of association, conditional on the sampling variances. The following algorithm summarizes the procedure:

Given *k* estimated effects, *t*_1_,*t*_2_,…,*t*_*k*_, and their variances, *v*_1_,*v*_2_,…,*v*_*k*_.

- For each replication, indexed *j*=1,2,…,*n*:
- Generate *k* estimated effects , where  and *δ* is the common effect size in the fixed effects model. Standardize these effects as described in the Background section, obtaining .
- Correlate  and *v*_1_,*v*_2_,…,*v*_*k*_ by computing Kendall’s tau, .
- Determine the intervals of rejection, e.g., by finding the percentiles based on the empirical distribution of . These intervals depend on the prescribed significance level *α*.
- Correlate the standardized effects, , based on the actual data, and the variances, *v*_1_,*v*_2_,…,*v*_*k*_, and compute Kendall’s tau, *τ*.
- Reject the null hypothesis of no publication bias if *τ* is within the rejection intervals or compute the *p*-value.

We denote this the adjusted Begg and Mazumdar test. The R code for the adjusted procedure is provided in Additional file 1. The intervals of rejection are estimated with errors. These errors may influence the results when testing for publication bias, and in order to minimize the errors, *n* should be large.

A drawback with this procedure is that we condition on the sampling variances, and we may possibly develop better methods if this is not done. In addition we assume that the estimated effect sizes are normally distributed. This may not always be the case in studies with a small sample size, and in particular, effect sizes are not normally distributed if the outcome is binary. Kendall’s tau is scale invariant. Hence the adjusted Begg and Mazumdar method will still work well if the variances are systematically underestimated. It should be noted that the simulation procedure itself in steps 1 and 2 does not depend on the observed values *t*_1_,*t*_2_,…,*t*_*k*_ of the random variables involved, but only on the fixed variances. Thus the procedure is not a bootstrap in the ordinary sense.

### Spearman’s rho versus Kendall’s tau as the measure of association

The Begg and Mazumdar test uses Kendall’s tau as the measure of association. It requires evaluation of the test statistic *S*=*C*−*D*, where *C* and *D* are the numbers of concordant and discordant pairs, respectively. This statistic can only take a finite number of distinct values, and the exact distribution of Kendall’s tau is discrete. The significance level may thus be incorrect, i.e., the actual significance level of the test may not equal the nominal value, especially for small values of *k*. This will be of less concern, however, as the value of *k* increases.

In order to improve the level, one may alternatively apply the mid-*p*-value [16], which deviates from the *p*-value in that only half the probability of the observed value of the test statistic is included in the tail. Additionally, one could employ the adjusted Begg and Mazumdar test based on Spearman’s rho. This statistic is defined as


where *d*_*i*_=*x*_*i*_−*y*_*i*_ is the difference between the ranks of observation *i* for the two variables. The distribution of Kendall’s tau converges faster towards the normal distribution than that of Spearman’s rho [17]. This may be one of the reasons why Begg and Mazumdar chose Kendall’s tau as a basis for their test procedure. However, having developed efficient computer programs for calculating the exact distribution of the rank correlation coefficient, this is no longer a valid argument in our setting. The exact distribution of Spearman’s rho is also discrete, but the test statistic can take more values compared to Kendall’s tau. Consequently, the actual significance level of Spearman’s rho converges faster towards the nominal level than Kendall’s tau, i.e., for smaller values of *k*.

### Simulation procedure

Simulations are needed in order to study the significance level of the Begg and Mazumdar test and to examine the operational characteristics of our new algorithm and compare to the original test. We apply the simulation procedure introduced by Begg and Mazumdar, and a detailed description is given in the following subsections.

#### Study selection

Begg and Mazumdar [6] assume that the sampling distribution of ***t*** is normal, i.e. *t*_*i*_∼*N*(*δ*,*v*_*i*_) and *t*_*i*_ is independent of *t*_*j*_ for *i*≠*j*. The component studies are designed to estimate a common effect size *δ* (we assume the fixed effects model), with variances depending on the sample sizes in the individual studies. All asymmetry in the simulated funnel plot is thus due to publication bias or chance; it cannot be explained by heterogeneity. The effect size, *t*, is in each case a summary estimate based on a data set of a certain size. Due to the Central Limit Theorem, *t* will possess an asymptotic normal distribution in most situations. According to Begg and Mazumdar [6], the assumption of normality is therefore reasonable.

When a particular value of *t*_*i*_ has been generated, the study is published (included in the meta-analysis) with probability given by an appropriate weight function. Begg and Mazumdar use different weight functions. The weight function depending on the *p*-value for the hypothesis that the true underlying effect is zero is defined as


with suitably defined constants *a* and *b*. This weight function is evaluated at  for one-sided selection, and  for two-sided selection. The absolute value was inadvertently left out in the article of Begg and Mazumdar. An alternative weight function based only on the assumption that the observed effect estimate determines the chance of publication is defined as


The main objective in conducting a meta-analysis is to estimate the true underlying effect, *δ*. This can be done using . The bias induced by the selection model, *β*, is defined as . The expected value is here defined with respect to the distribution of  after the selection of studies.

#### Scenarios

**Figure 2:**
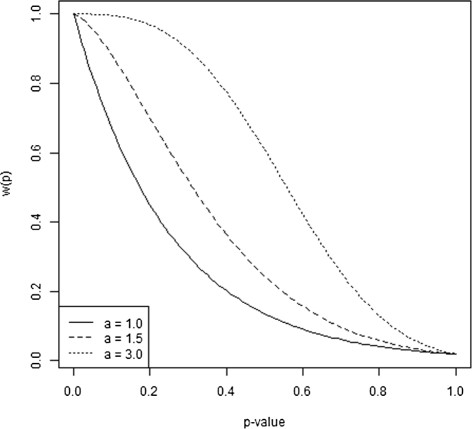
**Selection mechanism.** The weight function for selecting studies for inclusion in the meta-analysis as a function of the *p*-value. The value of *a* determines the selection strength (*a*=1.0: substantial selection strength, *a*=1.5: strong selection strength, *a*=3.0: moderate selection strength).

Following the scenarios of Begg and Mazumdar, we considered two values for *k*, the number of component studies in a meta-analysis, *k*=25 and *k*=75. For each simulated meta-analysis, the studies were generated in such a manner that after selection for inclusion in the meta-analysis, there were three equal-sized groups of studies with different variances. In each simulation, the middle group had a standardized variance of 1. When *k*=25, nine studies had variance 1. Two ranges of standardized variances were employed: large (*v*=0.1,1.0,10.0) and small (*v*=0.5,1.0,2.0). The range from the study with the smallest variance to the study with the largest variance was characterized by the logarithm to the base 10 of the ratio of these variances. The order of magnitude was 2 and 0.6 using Begg and Mazumdar’s choices of variances. The parameter *δ* reflecting the effect under study varied from zero, the null value, through 3.0 standard deviations from the null value. The scale was given in standard deviation units for the effect estimator for a study in the middle group having variance equal to 1.0. Publication bias in the meta-analysis was simulated using the selection functions presented in the previous subsection. Strong publication bias was obtained choosing *a*=1.5 and *b*=4.0, whereas *a*=3.0 and *b*=4.0 corresponded to moderate publication bias, see Figure 2. The choice of simulation parameters was justified by Begg and Mazumdar [6], and the same parameters were later used by Macaskill et al. [4] in their simulation studies. The selection model based on the *p*-value has also been described by Copas [18] and used by Preston et al. [19].

We generated each simulated meta-analysis in the following way. An effect size was randomly generated from a normal distribution having one of the variances under study. Its mean was the true, underlying effect. The probability of selection for inclusion in the meta-analysis was calculated by the relevant selection model. We chose the model based on the *p*-value. The decision to include or exclude this study in the meta-analysis was made based on a biased-coined randomization using the computed probability of publication. This procedure was repeated until a study with this first variance was included in the meta-analysis. We repeated the process, this time using a second variance under study. To avoid ties, we added a small term *ε*=0.0001 to each variance which had previously occurred in the simulation process of this particular meta-analysis. The process was continued until *k* studies had been selected with the required mixture of variances. The rank correlation test based on Kendall’s tau (or alternatively Spearman’s rho) was then calculated. The summary estimate of the true underlying effect was computed, and the number of studies required to generate *k* published studies was recorded. We repeated the entire process 5,000 times, a number originally chosen by Begg and Mazumdar. The two-sided empirical power and significance level of the test were calculated at the nominal 5% level. Since the error rates are expressed as proportions in a simulation study, the power estimates have a maximum standard error of 0.707*%*.

## Results

### Attained significance level and power of the original Begg and Mazumdar test based on Kendall’s tau

We first performed simulations in the situation without publication bias, in order to control the results of the Begg and Mazumdar method. The estimated significance level of their test for publication bias found by us is shown in Tables 1 and 2, in the same format as used by Begg and Mazumdar to report rejection rates. The first table gives the significance levels for small meta-analyses consisting of *k*=25 component studies. The second gives results for large meta-analyses that consist of *k*=75 component studies each.

**Table 1:** Significance level for the Begg and Mazumdar test for publication bias: Small meta-analyses*

	Level	
	[% selected for inclusion, bias]	
Range of variances	Large†	Small‡
Treatment effect (*δ*)		
.0	**1.72%**	**3.96%**
	[100%,.00]	[100%,.00]
.5	**1.82%**	**4.36%**
	[100%,.00]	[100%,.00]
1.0	**1.86%**	**4.30%**
	[100%,.00]	[100%,.00]
1.5	**1.90%**	**3.68%**
	[100%,.00]	[100%,.00]
2.0	**1.82%**	**3.58%**
	[100%,.00]	[100%, -.00]
2.5	**1.54%**	4.48%
	[100%,.00]	[100%,.00]
3.0	**1.74%**	**4.24%**
	[100%,.00]	[100%, -.00]

*k = 25 studies; nominal significance level 0.05.

†*v* = 0.1, 1.0, 10.0, ‡*v* = 0.5, 1.0, 2.0.

Values deviating significantly from the nominal level 5.00% over the 5000 simulations (using a 5% level in the binomial test) are typed in boldface.

**Table 2:** Significance level for the Begg and Mazumdar test for publication bias: Large meta-analyses*

	Level	
	[% selected for inclusion, bias]	
Range of variances	Large†	Small‡
Treatment effect (*δ*)		
.0	**1.76%**	**4.12%**
	[100%, -.00]	[100%,.00]
.5	**1.70%**	4.74%
	[100%,.00]	[100%, -.00]
1.0	**2.38%**	4.54%
	[100%,.00]	[100%,.00]
1.5	**1.96%**	**4.30%**
	[100%,.00]	[100%, -.00]
2.0	**1.60%**	**4.24%**
	[100%,.00]	[100%, -.00]
2.5	**1.88%**	**4.10%**
	[100%,.00]	[100%,.00]
3.0	**1.64%**	**4.22%**
	[100%,.00]	[100%,.00]

* *k*=75 studies; nominal significance level 0.05.

†*v* = 0.1, 1.0, 10.0, ‡*v* = 0.5, 1.0, 2.0.

Values deviating significantly from the nominal level 5.00% over the 5000 simulations (using a 5% level in the binomial test) are typed in boldface.

Our results confirm the impression that the attained significance level does not exceed the nominal one, but overall the deviations between the two values can be considerable. The range of variances is obviously an important factor influencing the significance level of the test. When the spread of variances is large (*v*=0.1,1.0,10.0), the significance level is roughly 0.02 or lower and deviates considerably from the nominal value of 0.05. When the range of variances is small (*v*=0.5,1.0,2.0), the significance level is approximately 0.04. The value of *k* does not seem to affect the level. We assessed the significance level also choosing *k*=300 and a large range of variances. The level is roughly the same as for *k*=25 and *k*=75, i.e. 0.02. The null hypothesis is rejected too infrequently. Due to these, perhaps unexpected, results, we believe a more thorough investigation of the operational characteristics is justified.

All simulations were undertaken using R [20]. The rank correlation test based on Kendall’s tau was calculated using the R function cor.test(), with the logical indicator exact set to NULL. An exact *p*-value for the hypothesis of no association is calculated in this function if there are less than 50 paired samples containing finite values and there are no ties. Otherwise, the asymptotically normal test statistic *z*, defined in the Methods section, is used. Similar simulations using the statistic *z* for small meta-analysis (*k*=25) were also carried out. The results confirm the observation that the overall significance level does not equal the nominal value if the asymptotic distribution of Kendall’s tau is used and is still below the nominal value. Thus, use of the exact or asymptotic distribution of Kendall’s tau does not explain the poor significance level when *k*=25.

In the simulation procedure presented by Begg and Mazumdar, the variances are treated as fixed constants. Additional simulations were carried out employing random variances drawn from a suitable distribution. As an example, for *k*=25 and *δ*=0 the level was only 0.0398 when drawing variances from an inverse gamma distribution with shape parameter 3.3 and rate parameter 300. The use of random variances does not remove the problem regarding the poor significance level. This is as expected because the fixed variance case has a strong resemblance to the Mann-Kendall trend test [21].

Power estimates were found employing the simulation procedure described in Methods. The simulations were restricted to one-sided selection depending on the *p*-value for the hypothesis that the true underlying effect is zero. The rank correlation test is not adequate for two-sided selection. At *δ*=0, the generated studies are symmetrically distributed around zero. Consequently, any selection function that is dependent on the two-sided *p*-value will result in a pattern in which the effect estimates and variances are uncorrelated [6].

The power of the Begg and Mazumdar test is shown in Tables 3 and 4. Best power is achieved for large meta-analyses where the spread of variance is large, the selection strength is strong and the treatment effect is small. As an example, the power is 0.99 when *k*=75, *v*=0.1,1.0,10.0, *a*=1.5 and *δ* = 0. When the selection strength is smaller (*a*=3), using the same values of the other parameters reduces the power to 0.88. When *k*=25, the power is moderate even for quite strong selection effects (*a*=1.5). It is estimated at 0.57 when *δ*=0 and *v*=0.1,1.0,10.0. The range of variances, which is inversely related to the spread of sample sizes, also has a substantial impact on the power. Reducing the range of variances in the latter situation (*v*=0.5,1.0,2.0), the power is only 0.22. The test is generally not powerful when the underlying treatment effect is far from the null value. However, this is not a major concern; in these situations there is only a small bias in the estimate of the treatment effect because the number of unpublished studies is lower.

**Table 3:** Power for the Begg and Mazumdar test for publication bias: Small meta-analyses*

	Power			
	[% selected for inclusion, bias]			
Selection strength	Strong**		Moderate***	
Range of variances	Large†	Small‡	Large†	Small‡
Treatment effect (*δ*)				
.0	57%	22%	33%	13%
	[36%,.34]	[37%,.74]	[57%,.25]	[57%,.54]
.5	51%	21%	23%	11%
	[54%,.16]	[52%,.54]	[74%,.09]	[73%,.34]
1.0	39%	16%	13%	8%
	[65%,.07]	[67%,.36]	[82%,.04]	[85%,.20]
1.5	27%	13%	9%	6%
	[72%,.05]	[80%,.23]	[87%,.02]	[92%,.10]
2.0	19%	8%	5%	5%
	[78%,.03]	[88%,.14]	[90%,.02]	[96%,.05]
2.5	12%	6%	3%	4%
	[82%,.02]	[93%,.07]	[93%,.01]	[98%,.03]
3.0	9%	5%	3%	4%
	[86%,.02]	[96%,.04]	[94%,.01]	[99%,.01]

*k = 25 studies; nominal significance level 0.05.

**a = 1.5, *** *a* = 3.0.

†*v* = 0.1, 1.0, 10.0, ‡*v* = 0.5, 1.0, 2.0.

**Table 4:** Power for the Begg and Mazumdar test for publication bias: Large meta-analyses*

	Power			
	[% selected for inclusion, bias]			
Selection strength	Strong**		Moderate***	
Range of variances	Large†	Small ‡	Large†	Small‡
Treatment effect (*δ*)				
.0	99%	61%	88%	38%
	[36%,.34]	[36%,.74]	[56%,.24]	[56%,.54]
.5	99%	59%	77%	31%
	[53%,.16]	[52%,.54]	[74%,.09]	[72%,.34]
1.0	94%	50%	54%	21%
	[64%,.07]	[67%,.36]	[82%,.04]	[84%,.19]
1.5	85%	35%	35%	12%
	[71%,.04]	[79%,.23]	[86%,.02]	[92%,.10]
2.0	71%	22%	21%	7%
	[77%,.03]	[88%,.13]	[90%,.02]	[96%,.05]
2.5	53%	12%	13%	5%
	[81%,.02]	[93%,.07]	[92%,.01]	[98%,.03]
3.0	40%	7%	8%	5%
	[85%,.02]	[96%,.04]	[94%,.01]	[99%,.01]

*k = 75 studies; nominal significance level 0.05.

**a = 1.5, ***a = 3.0.

†*v* = 0.1, 1.0, 10.0, ‡*v* = 0.5, 1.0, 2.0.

Additional simulations were carried out using a nominal level of 0.10. When the range of variances is large, the attained significance levels are roughly half the nominal, i.e., about 0.05. The significance level is estimated at 0.0498 when *k*=25 and *δ*=0. Employing a small spread of variances, the significance levels are about 0.09. When *k*=25 and *δ*=0, the empirical level is 0.090. As a consequence of increasing the significance level, the power of the test is also increased. Giving an example, the power equals 0.72 and 0.32 when *k*=25, *a*=1.5, *δ*=0 and the spread of variances is large and small, respectively.

Begg and Mazumdar justified the two ranges of variances used in their simulations by considering relevant values in published studies. In the context of meta-analysis, however, the variances take more than three different values, and their choices of variances do not represent a realistic distribution. Separate simulations confirmed that the distribution of the variances influences the results concerning the significance level and power, but these results are nevertheless not in conflict with our general assessments and conclusions. As an example, the simulation results when generating meta-analyses consisting of 25 component studies, of which 13 studies have the largest variance (*v*=10.0 or *v*=2.0), 9 have variance 1.0 and 3 have the lowest variance (*v*=0.1 or *v*=0.5), are consistent with the results in Table 3, see Additional file 2. The significance level was roughly 0.02 and 0.04 using the large and small range of variances, respectively.

### An explanation of the poor significance level

We compute the conditional covariance between two standardized effects given the variances. Straightforward calculations give


where *i*,*j*=1,2,…,*k* and *i*≠*j*.

We assume that the *k* pairs of random variables considered in a meta-analysis, (*t*_1_,*v*_1_),(*t*_2_,*v*_2_),…,(*t*_*k*_,*v*_*k*_), are independent and have the same bivariate distribution. As a consequence, *t*_*i*_ is independent of *t*_*j*_ given *v*_1_,*v*_2_,…,*v*_*k*_. It follows that the first term inside the square brackets equals zero. For the same reason,


The last term equals


It readily follows that


Because the variables  are standardized, we have


It has therefore been mathematically proven that the standardized treatment effects given the variances fail to be independent. As a result, the standardized treatment effects are not independent even if the variances are regarded as random variables. Furthermore, there is a correlation between the standardized effect sizes and sampling variances under the null hypothesis of no publication bias. It follows that the vectors ***t***^***∗***^ and ***v*** are not independent under the null hypothesis. The assumptions for using the rank correlation test are not strictly satisfied. We note that these results are independent of *k* and the distribution of ***t***^***∗***^, meaning that the problems still occur if the value of *k* is large and if the standardized effects are normally distributed.

Our calculations are consistent with a remark given by Begg [2], who claimed that a small correlation is produced because the empirical standardization is based on the *estimated* mean effect, which will be positively biased. However, when *k* = 25 and *v* = 0.1,1.0,10.0, then . The larger correlations are not negligible.

### Attained significance level and power for the adjusted Begg and Mazumdar test

The effect sizes are standardized by Begg and Mazumdar to obtain a set of estimates that can be assumed to be independent and identically distributed under the null hypothesis of no publication bias [4]. However, even after the standardization the assumptions for using the traditional rank correlation test are not met.

In the context of potential publication bias in meta-analysis, the adverse consequences of a false negative test are much more profound than those of a false positive test [2]. One should not conclude that publication bias is absent when it actually is present. It is essential to control or restrict the Type II error rate, and one should thus not be satisfied with a test that limits the significance level beneath the nominal value. Adjusting the significance level is a primary concern. Additionally, there are good reasons to choose a higher nominal significance level (e.g., *α*=0.10) than the conventional value 0.05. For a fixed sample size, increasing the significance level will increase the power of a given test method. Choosing a higher significance level is consequently a conservative measure in a meta-analysis setting.

We assessed the properties of the adjusted rank correlation test, first using Kendall’s tau as our test statistic. The adjusted interval of rejection for the test statistic was found in any particular situation with fixed variances using only the first two steps in the algorithm, choosing *n*=100,000. The area of acceptance was within the 2.5 and 97.5 percentiles from the empirical distribution of the simulated values of the measure of association. The nominal level of 0.05 was chosen to make results comparable to those of Begg and Mazumdar [6]. We used the same rejection intervals for all values of *δ*, the true underlying effect, that is, we only found new intervals for each value of *k* and each range of variances. The value of *δ* is unknown, but the significance level does not seem to depend on *δ* when the effect sizes are normally distributed (see Tables 1 and 2). Without loss of generality, one may thus choose *δ*=0. We then carried out the simulation procedure given by Begg and Mazumdar, employing the adjusted rejection intervals. In order to reduce the standard error, the entire process was now repeated 10,000 times. The maximum standard error from the latter simulations is then reduced from 0.707% to 0.500%.

When *k*=25, the attained significance level is 0.0542 and 0.0508 for *v*=0.1,1.0,10.0 and *v*=0.5,1.0,2.0, respectively. The algorithm obviously improves the Type I error rates. Table 5 presents the power of the adjusted test for *k*=25. As is to be expected, the Type II error rate is reduced compared to the original test introduced by Begg and Mazumdar. When *δ*=0, *v*=0.1,1.0,10.0 and *a*=1.5, the adjusted method increases the power to 0.73 compared to the original 0.57. When the selection strength is moderate (*a*=3), the corresponding numbers are 0.48 and 0.33 for the adjusted and original Begg and Mazumdar test, respectively. In cases where the range of variances is small, the attained significance level is close to the nominal value for the Begg and Mazumdar test. Consequently, there is only a minor improvement in the Type II error rate when correcting the significance level. Nevertheless, the adjusted method is clearly preferable to the original test. Similar conclusions are drawn when the meta-analyses comprise 75 studies, and the results of the power simulations are shown in Additional file 3. The attained significance level is 0.0501 and 0.0507 for *v*=0.1,1.0,10.0 and *v*=0.5,1.0,2.0, respectively.

**Table 5:** Power for the adjusted Begg and Mazumdar test based on Kendall’s tau: Small meta-analyses*

	Power			
	[% selected for inclusion, bias]			
Selection strength	Strong**		Moderate***	
Range of variances	Large†	Small‡	Large†	Small‡
Treatment effect (*δ*)				
.0	73%	24%	48%	16%
	[36%,.34]	[37%,.74]	[57%,.25]	[57%,.54]
.5	69%	23%	37%	14%
	[54%,.16]	[52%,.54]	[74%,.09]	[73%,.35]
1.0	56%	20%	25%	10%
	[65%,.07]	[67%,.37]	[82%,.04]	[85%,.20]
1.5	44%	15%	17%	7%
	[72%,.05]	[80%,.23]	[87%,.02]	[92%,.10]
2.0	32%	10%	12%	6%
	[78%,.03]	[88%,.13]	[90%,.02]	[96%,.05]
2.5	24%	7%	9%	5%
	[82%,.02]	[93%,.07]	[93%,.01]	[98%,.03]
3.0	19%	6%	7%	5%
	[86%,.02]	[97%,.04]	[94%,.01]	[99%,.01]

*k = 25 studies; nominal significance level 0.05.

**a = 1.5, *** *a* = 3.0.

†*v* = 0.1, 1.0, 10.0, ‡*v* = 0.5, 1.0, 2.0.

**Figure 3:**
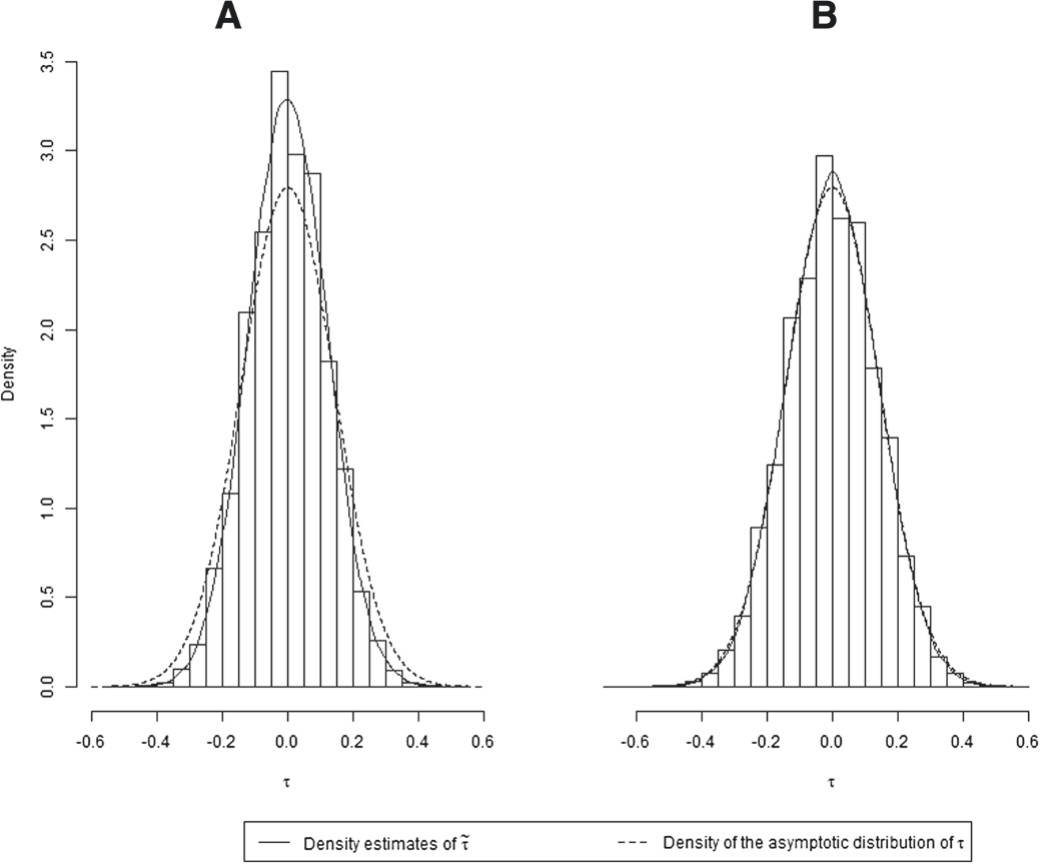
**Histograms of the simulated distribution of**  **under the null hypothesis along with kernal density estimates.** These are compared to the density of the asymptotic distribution of Kendall’s tau. **A**: small meta-analyses (*k*=25) and a large range of variances (*v*=0.1,1.0,10.0); **B**: small meta-analyses (*k*=25) and a small range of variances (*v*=0.5,1.0,2.0).

Figure 3 shows histograms of the simulated distribution of  under the null hypothesis for *k*=25, together with kernel density estimates. These are compared to the density of the asymptotic distribution of Kendall’s tau. The histograms are based on 100,000 simulated values of , and the kernel density estimates were computed applying the R function density [20]. When there is a large spread of variances (Figure 3A), the simulated distribution of  clearly deviates from the theoretical. This difference decreases when the range of variances is small (Figure 3B). This result supports our findings in Tables 1 and 3; when the attained level of the Begg and Mazumdar test is close to the nominal, it performs equally well as the adjusted test.

Additional simulations were performed choosing *a*=1.0 when *k*=25 for both the original and the adjusted Begg and Mazumdar test. This value of *a* generates even stronger selection bias than *a*=1.5, see Figure 2. In line with earlier findings, the power was increased for both methods with the substantial publication bias, and the adjusted test still gave the best Type II error rates. The results are shown in Additional file 4.

As already explained, it may be reasonable to choose a higher significance level than 0.05, and simulations were carried out for the adjusted method at a 0.10 significance level. These additional simulations were also performed when the meta-analyses comprised *k*=25 studies, this value of *k* being more relevant than *k*=75 in clinical and epidemiological settings. The trade-off effect reduces the Type II error rate at the expense of the Type I error rate. The results are shown in Table 6. The power is still poor when the spread of variances is small, and the power estimates are below 0.36. However, the test gives respectable power in the remaining situations, at least when the treatment effect is close to the null value. At *a*=1.5, the power ranges between 0.83 and 0.30 for all treatment effects. The attained significance level was close to the nominal value and was estimated at 0.1064 and 0.1017 when *v*=0.1,1.0,10.0 and *v*=0.5,1.0,2.0, respectively.

**Table 6:** Power for the adjusted Begg and Mazumdar test based on Kendall’s tau: Small meta-analyses*

	Power			
	[% selected for inclusion, bias]			
Selection strength	Strong**		Moderate***	
Range of variances	Large†	Small‡	Large†	Small‡
Treatment effect (*δ*)				
.0	83%	36%	63%	23%
	[36%,.34]	[37%,.74]	[57%,.25]	[57%,.54]
.5	80%	34%	53%	22%
	[54%,.16]	[52%,.54]	[74%,.09]	[73%,.34]
1.0	70%	30%	39%	17%
	[65%,.07]	[67%,.36]	[82%,.04]	[85%,.20]
1.5	59%	23%	27%	13%
	[72%,.05]	[80%,.23]	[87%,.03]	[92%,.11]
2.0	48%	18%	21%	10%
	[78%,.03]	[88%,.13]	[90%,.02]	[96%,.05]
2.5	37%	13%	17%	10%
	[82%,.02]	[93%,.08]	[93%,.01]	[98%,.03]
3.0	30%	10%	14%	10%
	[86%,.02]	[96%,.04]	[94%,.01]	[99%,.01]

*k = 25 studies; nominal significance level 0.10.

**a = 1.5, ***a = 3.0.

†*v* = 0.1, 1.0, 10.0, ‡*v* = 0.5, 1.0, 2.0.

Many meta-analyses include much less than 25 studies. For that reason, we performed additional simulations in the Begg and Mazumdar setting to assess the actual significance level of the adjusted test as *k* decreases, still using Kendall’s tau as our test statistic. The results depend on the range of variances. When the spread of variances is large (*v*=0.1,1.0,10.0), a reasonably correct significance level is attained for *k*=16. For a small spread of variances (*v*=0.5,1.0,2.0), the correct significance level is achieved for *k*≥18. The power of the different tests for publication bias is generally of great concern. This problem will not decrease for small values of *k*. The best power is, as usual, attained when strong selection bias is present and *δ*=0. When, in addition, the range of variance is large and *k*=16, the simulated power of the test is 0.55. With a small range of variances and *k*=18, the test achieves a power of 0.19. The estimated significance levels are 0.0481 and 0.0513, respectively.

What if we use Spearman’s rho instead of Kendall’s tau as the measure of association? Additional simulations then demonstrate that *k*≥10 is sufficient to achieve an acceptable significance level when the range of variances is large (*v*=0.1,1.0,10.0). For small spread of variances (*v*=0.5,1.0,2.0), a reasonably correct significance level cannot be guaranteed for *k*<11. The power attained given strong selection bias and *δ*=0 is in each case 0.33 and 0.13, respectively. The attained significance levels are 0.0492 and 0.0528. The results of the adjusted test based on Spearman’s rho in the Begg and Mazumdar simulation setting are presented in Tables 7 and 8 at a nominal significance level of 0.05 and 0.10, respectively. Both tables display results for small meta-analyses (*k*=25). We see that the power estimates are similar to the ones attained using Kendall’s tau. The attained significance levels are approximately equal to the nominal value in all cases. At the nominal 0.05 level, it is estimated at 0.0483 when *v*=0.1,1.0,10.0 and at 0.0504 when *v*=0.5,1.0,2.0. The corresponding values at the nominal 10% level are 0.1012 and 0.0995.

**Table 7:** Power for the adjusted Begg and Mazumdar test based on Spearman’s rho: Small meta-analyses*

	Power			
	[% selected for inclusion, bias]			
Selection strength	Strong**		Moderate***	
Range of variances	Large†	Small‡	Large†	Small‡
Treatment effect (*δ*)				
.0	74%	24%	52%	16%
	[36%,.34]	[37%,.74]	[57%,.25]	[57%,.54]
.5	69%	23%	39%	14%
	[54%,.16]	[52%,.54]	[74%,.09]	[73%,.34]
1.0	57%	20%	26%	10%
	[65%,.07]	[67%,.37]	[82%,.04]	[85%,.20]
1.5	44%	15%	17%	7%
	[72%,.05]	[80%,.23]	[87%,.03]	[92%,.10]
2.0	34%	10%	12%	5%
	[78%,.03]	[88%,.13]	[90%,.02]	[96%,.05]
2.5	25%	7%	9%	5%
	[82%,.02]	[93%,.07]	[93%,.01]	[98%,.03]
3.0	19%	6%	8%	5%
	[86%,.02]	[97%,.04]	[94%,.01]	[99%,.01]

*k = 25 studies; nominal significance level 0.05.

**a = 1.5, ***a = 3.0.

†*v* = 0.1, 1.0, 10.0, ‡*v* = 0.5, 1.0, 2.0.

**Table 8:** Power for the adjusted Begg and Mazumdar test based on Spearman’s rho: Small meta-analyses*

	Power			
	[% selected for inclusion, bias]			
Selection strength	Strong**		Moderate***	
Range of variances	Large†	Small‡	Large†	Small‡
Treatment effect (*δ*)				
.0	84%	36%	64%	25%
	[36%,.34]	[36%,.74]	[57%,.25]	[57%,.54]
.5	80%	35%	53%	22%
	[54%,.16]	[52%,.54]	[74%,.09]	[73%,.34]
1.0	70%	31%	38%	18%
	[65%,.07]	[67%,.37]	[82%,.04]	[85%,.20]
1.5	58%	25%	27%	13%
	[72%,.05]	[80%,.23]	[87%,.03]	[92%,.10]
2.0	47%	18%	22%	11%
	[78%,.03]	[88%,.13]	[90%,.02]	[96%,.05]
2.5	37%	13%	17%	10%
	[82%,.02]	[93%,.08]	[93%,.01]	[98%,.03]
3.0	30%	11%	14%	10%
	[86%,.02]	[97%,.04]	[94%,.01]	[99%,.01]

*k = 25 studies; nominal significance level 0.10.

**a = 1.5, ***a = 3.0.

†*v* = 0.1, 1.0, 10.0, ‡*v* = 0.5, 1.0, 2.0.

### Examples

We compare the new, adjusted test to that of Begg and Mazumdar, applying two examples from the literature [22–24]. These examples have previously been used by Begg and Mazumdar [6] and Begg [2], respectively, in illustration of rank correlation tests for publication bias; the potential problem of publication bias was recognized in both examples and was supported by the funnel plots in their papers. The raw data can be found in the original articles [22, 23] and are also presented by Begg and Mazumdar [6] and Begg [2].

**Example 1** In the first meta-analysis, Cottingham and Hunter [22] studied the association between *Chlamydia trachomatis* and oral contraceptive use. The analysis was based on 29 case-control studies and two prospective studies. Begg and Mazumdar excluded the prospective studies, and they are thus also excluded from our example. In order to correct for tied values in the dataset, we applied the R function Kendall [25] to compute the rank correlation based on Kendall’s tau and its *p*-value when using the traditional rank correlation test. For the modified tests, we used the R function cor [20]. Applying the Begg and Mazumdar test, Kendall’s tau equals 0.21, and we obtain a two-sided *p*-value of 0.115. Hence we do not reject the hypothesis of no publication bias, using a nominal significance level of 0.10. This value deviates from the result reported by Begg and Mazumdar (*p*-value of 0.08), and only some of this discrepancy can be explained by Begg and Mazumdar not accounting for ties. The adjusted tests based on Kendall’s tau and Spearman’s rho give mid-*p*-values of 0.081 and 0.094. The value of Spearman’s rho is 0.29. Both results indicate publication bias, which is in agreement with the visual assessment of the funnel plot [6].

**Example 2** The second example [23, 24] is a review of randomized experiments on the effects of teacher expectancy on pupil IQ. A total number of 19 studies were included in the analysis. The Begg and Mazumdar test, again using the R function Kendall to account for ties, gives a two-sided *p*-value of 0.080. The rank correlation coefficient equals 0.30. Applying the adjusted method based on Kendall’s tau, we obtain a mid-*p*-value of 0.067, and the adjusted method based on Spearman’s rho gives a mid-*p*-value of 0.063. Spearman’s rho equals 0.43. The results are consistent with the findings of the Begg and Mazumdar test and the visual interpretation of the funnel plot [2], although a bit more conservative in the meta-analysis setting.

## Discussion

Although the test introduced by Begg and Mazumdar is well known and often cited in published work (Figure 1), it has serious drawbacks. To our knowledge, these disadvantages have not been adequately discussed in the literature. The significance level does not equal the nominal level, the main reason being the non-zero conditional covariance between the standardized treatment effects given the variances. The adverse consequences are difficult to ignore. We propose improvements of the Begg and Mazumdar test when the outcome is assumed to be normally distributed. Although it is more computer intensive, we recommend using the adjusted Begg and Mazumdar method when assuming fixed effects meta-analysis, which attains significance levels that equal the nominal value. This reduces the Type II error.

When outcomes are binary, however, there are tests particularly designed for handling the additional difficulties that arise in this situation [5, 13, 14]. Sterne et al. [26] give an overview of these tests. Nevertheless, results of a recently published simulation study [27] indicate that even these tests might not perform well in many situations.

Overall, we advocate the use of the adjusted method based on Spearman’s rho; it makes it easier to control the Type I error rate for small values of *k* compared to the adjusted test based on Kendall’s tau. Sterne at al. [26] do not recommend tests for funnel plot asymmetry when there are fewer than ten studies in the meta-analysis, due to the fact that test power is usually too low to distinguish chance from real asymmetry. Our findings support this reasoning. An additional argument is that we are not able to control the significance level for values of *k* smaller than ten. However, if Kendall’s tau is used as a basis for the test instead of Spearman’s rho, the minimum number of studies should be at least 16 in order to control the significance level. Our study has not evaluated situations with heterogeneity in effects between studies; in these situations the minimum number of studies required may be substantially higher than ten.

Although the improved tests are more powerful than the test by Begg and Mazumdar, their general power is still limited, particularly for moderate amounts of bias and when the total number of studies included in the meta-analysis is typical of standard practice in medical applications. Tests for small-study effects should routinely be performed prior to conducting a meta-analysis. Nevertheless, it is important not to rule out the possibility of small-study effects when the tests do not produce significant results. Even when evidence of small-study effects is found in the meta-analysis, careful consideration should be given to possible explanations, e.g., publication bias and heterogeneity.

There are some limitations to our study that need to be addressed. We have only regarded the fixed effects model, i.e. no heterogeneity is assumed between the component studies in the meta-analysis. This is consistent with the original Begg and Mazumdar formulation [6]. The model is therefore sufficient for demonstrating the adverse consequences of their test. To fully understand the properties of our proposed method it should also be assessed in a random effects model. Such models assign larger weights to small studies, which are thus less likely to be trivialized, and this may affect the behaviour of our test. Nevertheless, such an assessment requires a whole new simulation study and is beyond the scope of this paper.

In addition, a more realistic distribution of the component studies in the meta-analyses should be used in new simulations. Furthermore, the selection function considered in our paper is very simplistic and depends only on the *p*-value. We believe it is sufficient for illustrating the limitations of the Begg and Mazumdar test and for introducing our simulation procedure. Nevertheless, in a new simulation study more sophisticated selection models, using more of the available information, should be employed. Baker and Jackson [28] and Bowden et al. [29] propose methods involving information on journal impact factor and publishing author.

The adjusted test corrects the significance level. It therefore forms a better basis for comparing the different test statistics introduced in the literature, e.g., those presented by Egger et al. [8] and Macaskill et al. [4]. Several papers have compared different methods (e.g. [4, 30]). However, as several tests fail to give the correct Type I error, it is difficult to evaluate and compare the performance of the test statistics. The general idea behind our proposed simulation method, involving the empirical distribution, is of course not novel. Nevertheless, we have not seen this simple method used in the literature of publication bias. The procedure may not only be applied to the rank correlation tests but also to other test statistics introduced in this field. New simulation studies should be conducted comparing the performance of different statistics when we are able to control their significance level. Unfortunately, such studies are also beyond the scope of our article.

The methods considered in this paper test for publication bias in meta-analysis. Several authors address the issue of how to proceed if a test for publication bias is significant. Duval and Tweedie [31, 32] introduce an iterative non-parametric method to provide an adjusted estimate of the treatment effect, known as the trim-and-fill method. The method is based on the symmetry property of the funnel graph and is a useful supplement to the methods discussed in this paper. Bürkner and Doebler [27] also suggest using the trim-and-fill method as an option for handling publication bias in meta-analysis when outcomes are binary. The Copas selection model [33] is a parametric statistical model which combines the random effects model with a selection model. However, the selection model requires a sensitivity analysis. Rücker et al. [34] state that the trim-and-fill method, as well as the Copas selection model, may not fully eliminate bias. Regression-based methods [30, 34, 35] may be promising alternatives, as they are not formed on the basis of a specific selection model and are easier to implement. A further discussion of this topic will, however, not be given here.

## Conclusion

We showed in simulations that the significance level of the rank correlation test introduced by Begg and Mazumdar often deviates considerably from the nominal level. Additionally, we proved that the assumptions for using a rank correlation test are not met. A modified rank correlation test which is based on the simulated distribution of the estimated measure of association, preferably Spearman’s rho, conditional on sampling variances, improves the error rates. This should thus be chosen over the conventional Begg and Mazumdar test in the case of normally distributed outcomes when testing for publication bias assuming fixed effects meta-analysis.

## Electronic supplementary material

Additional file 1: **R code for the adjusted Begg and Mazumdar test based on Kendall’s tau.** The R code provides the mid-*p* value for the adjusted Begg and Mazumdar test based on Kendall’s tau. The code shows how the algorithm in the Methods section can be implemented. (PDF 52 KB)

Additional file 2: **Power for the original Begg and Mazumdar test, employing a somewhat more realistic distribution of the variances for small meta-analyses.** The meta-analyses are generated consisting of 13 component studies having the largest variance (*v*=10.0 or *v*=2.0), 9 component studies with variance 1.0 and 3 component studies with the lowest variance (*v*=0.1 or *v*=0.5). (PDF 53 KB)

Additional file 3: **Power for the adjusted Begg and Mazumdar test based on Kendall’s tau for large meta-analyses (** ***k*** ***=*** **75).** (PDF 53 KB)

Additional file 4: **Power for both the original Begg and Mazumdar test and the adjusted procedure when applying even stronger selection strength (** ***a*** ***=*** **1.0) for small meta-analyses (** ***k*** ***=*** **25).** (PDF 53 KB)
