# Supplementary material for: Improving the error rates of the Begg and Mazumdar test for publication bias in fixed effects meta-analysis
Source: BMC Med Res Methodol. 2014 Sep 22;14:109. doi: 10.1186/1471-2288-14-109 (PMC4193136; doi:10.1186/1471-2288-14-109)
Supplement: Supplementary file 1 — Additional file 1: R code for the adjusted Begg and Mazumdar test based on Kendall’s tau. The R code provides the mid-p value for the adjusted Begg and Mazumdar test based on Kendall’s tau. The code shows how the algorithm in the Methods section can be implemented. (PDF 52 KB) [file 12874_2014_1124_MOESM1_ESM.pdf]

Additional file 1 — R code for the adjusted Begg and Mazumdar test based on Kendall's tau

The R code provides the mid-p value for the adjusted Begg and Mazumdar test based on Kendall's tau. The code shows how the algorithm in the Methods section can be implemented.

```
adjustedBeggMazumdar <- function(t, v, n){

    # Returns the two-sided mid-p value for the adjusted
    # Begg and Mazumdar test based on Kendall's tau

    # Given a meta- analysis consisting of .k studies
    # t is a numeric vector of the effect estimates attained from the .k studies
    # v is a numeric vector of the variances attained from the .k studies
    # n gives the number of replications

    # Number of studies in the meta-analyses
    .k <- length(t)

    # -- Simulations following the proposed algorithm --

    # Step 1(a): Generate the standardized estimated effects
    .t.tilde <- matrix(rnorm(.k*n,0,sqrt(v)),.k,n)
    .t.tilde.bar <- apply(.t.tilde,2,function(x) sum(v^(-1)*x)/sum(v^(-1)))
    .v.star <- v-(sum(v^(-1)))^(-1)
    .t.tilde.star <- t(t(.t.tilde)-.t.tilde.bar)/.v.star^0.5

    # Step 1(b): Correlate the generated standardized effects and variances,
    # using, for example, Kendall's tau
    .tau.tilde <- apply(.t.tilde.star,2,function(x) Kendall(x,v)$tau)

    # Step 2: Determine the intervals of rejection.
    # Not needed when computing the mid-p value

    # Step 3: Correlate the standardized effects, based on the actual data,
    # and variances
    .t.bar <- sum(v^(-1)*t)/sum(v^(-1))
    .t.star <- (t-.t.bar)/.v.star^0.5
    .tau <- Kendall(.t.star,v)$tau

    # Step 4: Compute mid-p value
    if(.tau < median(.tau.tilde)){
        mid_p <- (length(which(.tau.tilde<.tau)) +
                    length(which(.tau.tilde==.tau))/2)*2/length(.tau.tilde)
    }else if(.tau>median(.tau.tilde)){
        mid_p <- (length(which(.tau.tilde>.tau)) +
                    length(which(.tau.tilde==.tau))/2)*2/length(.tau.tilde)
    }else if(.tau==median(.tau.tilde)) mid_p <- 1

    return(mid_p)

}
```
