# Supplementary material for: Improving the error rates of the Begg and Mazumdar test for publication bias in fixed effects meta-analysis
Source: BMC Med Res Methodol. 2014 Sep 22;14:109. doi: 10.1186/1471-2288-14-109 (PMC4193136; doi:10.1186/1471-2288-14-109)
Supplement: Supplementary file 2 — Additional file 2: Power for the original Begg and Mazumdar test, employing a somewhat more realistic distribution of the variances for small meta-analyses. The meta-analyses are generated consisting of 13 component studies having the largest variance (v=10.0 or v=2.0), 9 component studies with variance 1.0 and 3 component studies with the lowest variance (v=0.1 or v=0.5). (PDF 53 KB) [file 12874_2014_1124_MOESM2_ESM.pdf]

Additional file 2 — Power for the Begg and Mazumdar test for publication bias, employing a somewhat more realistic variance distribution: Small meta-analyses*

	Power [% selected for inclusion, bias]			
Selection strength	Strong**		Moderate***	
Range of variances	Large†	Small‡	Large†	Small‡
Treatment effect (δ)				
.0	40% [37%, .47]	17% [37%, .86]	25% [57%, .34]	11% [57%, .63]
.5	39% [48%, .28]	16% [50%, .67]	22% [69%, .17]	10% [71%, .43]
1.0	35% [56%, .18]	14% [63%, .48]	17% [76%, .11]	9% [82%, .28]
1.5	33% [64%, .13]	12% [75%, .33]	12% [82%, .07]	7% [90%, .16]
2.0	26% [70%, .10]	9% [85%, .22]	8% [85%, .05]	5% [95%, .09]
2.5	20% [74%, .07]	6% [91%, .14]	6% [89%, .03]	4% [97%, .05]
3.0	15% [79%, .06]	5% [95%, .08]	5% [91%, .03]	5% [99%, .02]

* $k = 25$ studies; nominal significance level 0.05
** $a = 1.5$, *** $a = 3.0$
† $v = 0.1, 1.0, 10.0$, ‡ $v = 0.5, 1.0, 2.0$
For each range of variances, 13 studies have the largest variance, 9 studies have variance 1.0 and 3 studies have the smallest variance
