# Supplementary material for: Improving the error rates of the Begg and Mazumdar test for publication bias in fixed effects meta-analysis
Source: BMC Med Res Methodol. 2014 Sep 22;14:109. doi: 10.1186/1471-2288-14-109 (PMC4193136; doi:10.1186/1471-2288-14-109)
Supplement: Supplementary file 3 — Additional file 3: Power for the adjusted Begg and Mazumdar test based on Kendall’s tau for large meta-analyses ( k = 75). (PDF 53 KB) [file 12874_2014_1124_MOESM3_ESM.pdf]

Additional file 3 — Power for the adjusted Begg and Mazumdar test based on Kendall's tau: Large meta-analyses*

	Power [% selected for inclusion, bias]			
Selection strength	Strong**		Moderate***	
Range of variances	Large†	Small‡	Large†	Small‡
Treatment effect (δ)				
.0	100% [36%, .34]	63% [36%, .74]	95% [56%, .25]	40% [56%, .54]
.5	99% [53%, .16]	62% [52%, .54]	88% [74%, .09]	34% [73%, .34]
1.0	98% [64%, .07]	54% [67%, .36]	71% [82%, .04]	24% [85%, .20]
1.5	93% [71%, .04]	40% [79%, .22]	52% [86%, .02]	14% [92%, .10]
2.0	84% [77%, .03]	25% [88%, .13]	36% [90%, .02]	8% [96%, .05]
2.5	71% [81%, .02]	14% [93%, .07]	25% [92%, .01]	6% [98%, .03]
3.0	56% [85%, .02]	8% [96%, .04]	18% [94%, .01]	5% [99%, .01]

* $k = 75$ studies; nominal significance level 0.05
** $a = 1.5$, *** $a = 3.0$
† $v = 0.1, 1.0, 10.0$, ‡ $v = 0.5, 1.0, 2.0$
