# Supplementary material for: Improving the error rates of the Begg and Mazumdar test for publication bias in fixed effects meta-analysis
Source: BMC Med Res Methodol. 2014 Sep 22;14:109. doi: 10.1186/1471-2288-14-109 (PMC4193136; doi:10.1186/1471-2288-14-109)
Supplement: Supplementary file 4 — Additional file 4: Power for both the original Begg and Mazumdar test and the adjusted procedure when applying even stronger selection strength ( a = 1.0) for small meta-analyses ( k = 25). (PDF 53 KB) [file 12874_2014_1124_MOESM4_ESM.pdf]

Additional file 4 — Additional power simulations for even stronger selection strength*: Small meta-analyses**

	Power [% selected for inclusion, bias]			
Method	Begg and Mazumdar		Adjusted Begg and Mazumdar	
Range of variances	Large†	Small‡	Large†	Small‡
Treatment effect (δ)				
.0	62% [25%, .38]	24% [25%, .84]	79% [25%, .38]	28% [25%, .84]
.5	65% [40%, .21]	24% [39%, .66]	80% [41%, .21]	29% [39%, .66]
1.0	56% [51%, .10]	23% [53%, .49]	73% [51%, .10]	27% [53%, .49]
1.5	44% [59%, .07]	20% [67%, .34]	64% [59%, .07]	22% [67%, .34]
2.0	33% [66%, .05]	14% [79%, .21]	52% [66%, .05]	17% [78%, .21]
2.5	24% [72%, .03]	9% [86%, .14]	43% [72%, .04]	11% [87%, .13]
3.0	18% [77%, .02]	6% [92%, .08]	34% [76%, .02]	7% [92%, .08]

* $a = 1.0$
** $k = 25$ studies; nominal significance level 0.05
† $v = 0.1, 1.0, 10.0$, ‡ $v = 0.5, 1.0, 2.0$
Both tests are based on Kendall's tau
